# Plant Antimicrobial Peptides as Potential Tool for Topic Treatment of Hidradenitis Suppurativa

**DOI:** 10.3389/fmicb.2021.795217

**Published:** 2021-12-13

**Authors:** Carlos André dos Santos-Silva, Paola Maura Tricarico, Lívia Maria Batista Vilela, Ricardo Salas Roldan-Filho, Vinícius Costa Amador, Adamo Pio d’Adamo, Mireli de Santana Rêgo, Ana Maria Benko-Iseppon, Sergio Crovella

**Affiliations:** ^1^Institute for Maternal and Child Health – IRCCS Burlo Garofolo, Trieste, Italy; ^2^Departamento de Genética, Centro de Biociências, Universidade Federal de Pernambuco, Recife, Brazil; ^3^Department of Medical Surgical and Health Sciences, University of Trieste, Trieste, Italy; ^4^Biological Science Program, Department of Biological and Environmental Sciences, College of Arts and Sciences, Qatar University, Doha, Qatar

**Keywords:** antimicrobial peptides, plants, hidradenitis suppurativa, infections, antibiotics, bioinformatics

## Abstract

Among chronic skin autoinflammatory diseases, Hidradenitis Suppurativa (HS) stands out for its chronicity, highly variable condition, and profound impact on the patients’ quality of life. HS is characterized by suppurative skin lesions in diverse body areas, including deep-seated painful nodules, abscesses, draining sinus, and bridged scars, among others, with typical topography. To date, HS is considered a refractory disease and medical treatments aim to reduce the incidence, the infection, and the pain of the lesions. For this purpose, different classes of drugs, including anti-inflammatory molecules, antibiotics and biological drugs are being used. Antimicrobial peptides (AMPs), also called defense peptides, emerge as a new class of therapeutic compounds, with broad-spectrum antimicrobial action, in addition to reports on their anti-inflammatory, healing, and immunomodulating activity. Such peptides are present in prokaryotes and eukaryotes, as part of the innate eukaryotic immune system. It has been proposed that a deregulation in the expression of AMPs in human epithelial tissues of HS patients may be associated with the etiology of this skin disease. In this scenario, plant AMPs stand out for their richness, diversity of types, and broad antimicrobial effects, with potential application for topical systemic use in patients affected by HS.

## Introduction

Hidradenitis suppurativa (HS) is a chronic disease characterized by recurrent, painful, deep-seated, rounded nodules and abscesses of apocrine gland-bearing skin. HS is a chronic disease, late diagnosed with a profound impact on the patients’ quality of life. HS is characterized by recurrent skin lesions and may develop into subcutaneous extension leading to sinus and fistula tracts formation. These painful lesions represent the main problem for the patients’ quality of life, being periodic and not always responding well to a topic antibiotic therapy, with the onset of antibiotic resistance (Revuz, 2009; O’Neal et al., 2020).

The resistance developed by microorganisms is an inevitable result of the fragile balance between bacteria and antibiotics. However, bacteria have infinitely more opportunities to gain resistance than humans who must create new antimicrobial agents due to generation time (Huttner et al., 2013). Over the years, humans have used nature to meet their basic needs, as well as seeking medicines to treat a wide spectrum of diseases. Plants seem to be an undeniable source of bioactive molecules to fight various diseases, including multi-resistant bacteria (Benko-Iseppon et al., 2010; Porto et al., 2018a). These medicinal properties have been investigated in recent years, with emphasis on plant antimicrobial peptides (AMPs); AMPs are low molecular mass defense molecules with a vast and potent inhibitory activity against a wide range of organisms (Santos-Silva et al., 2020).

Recent interest in AMPs for therapeutic applications has been developed due to the need for new antibiotics since bacterial resistance to traditional molecules arose (Su et al., 2020). Presumably, bacteria have been exposed to AMPs for millions of years and, except for a few species (such as *Burkholderia* spp.), generalized resistance has not been reported (Loutet, 2011). Notably, the development of resistance against AMPs occurred to a much lesser degree, as there could not be a general mechanism of resistance to AMPs, requiring multiple pathways for this, for example, attacking multiple hydrophobic and/or polyanionic targets (Fjell et al., 2012). Therefore, AMPs emerged as potential candidates in the search for new therapeutic agents against bacterial resistance (Liu et al., 2016; Sun et al., 2019).

This review brings an up-to-date overview of the general knowledge of HS, focusing on the AMPs role as potential molecules for HS patients’ topic treatment.

## Hidradenitis Suppurativa – General Features

Hidradenitis suppurativa, also known as Acne Inversa for its suppurative lesions affecting inverse areas, is a chronic autoinflammatory skin disease characterized by a complex clinical phenotype with variable degrees of severity. HS can be generally described as an inflammatory persistent, enduring disease characterized by the presence of inflammatory lesions mainly found in the areas of the body in which apocrine glands are localized. The inflamed suppurative lesions may cause constant pain, thus dramatically impacting patients’ quality of life (Preda-Naumescu et al., 2021).

Hidradenitis suppurativa was first described in 1854 by the French surgeon Verneuil (1854); despite this, the disease is still not completely understood and misdiagnosed (Rick et al., 2021). In fact, the exact etiology is not yet entirely unraveled. However, it is widely accepted that HS is a complex disease with a multifactorial etiology that involves complex interactions between genetic factors, immune dysregulation, bacterial infections, and environmental risk factors (Schell et al., 2021). The influence of genetic variations has been observed in about one-third of HS patients and are mainly reported in the genes of the gamma-secretase complex, namely *NCSTN*, *PEN*-1, and *PSENEN* (van Straalen et al., 2020).

Immune dysregulation, in particular aberrant activation of the immune system involving different signaling pathways resulting in dysregulation of the release of various anti- or pro-inflammatory molecules, e.g., TNF-α, IL-1β, AMPs, has been reported in HS (Jiang et al., 2021). The interaction between immune dysregulation and skin bacteria plays a central role in the disease, promoting the chronicity of HS (Marasca et al., 2020); despite this evidence, the microbiological aspects of HS are not yet fully described. It has been hypothesized that cutaneous dysbiosis, characterized by the presence of pathogens and commensals bacteria, plays a role in HS (Guet-Revillet et al., 2017). Smoking and obesity are the two of the most frequent environmental risk factors reported in HS (Ingram, 2020).

To date, the frequency of HS is relatively high, reaching 1% in the European population (Ingram, 2020), i.e., the same as celiac disease. Low HS frequencies have been reported in several countries, but they are considered the result of the underdiagnosis of the disease. In fact, HS is an ailment with a long delay between its occurrence and the clinical diagnosis, reflecting the unequal worldwide distribution of dermatological resources as well as dedicated centers, also depending upon socio-economic issues. Moreover, ethnicity is also supposed to play a role in the distribution of HS worldwide (Reeder et al., 2014; Lee D.E. et al., 2017; Sachdeva et al., 2021).

Interestingly, based on 16 studies performed in Scandinavia, Western Europe, United States, and Australia, a recent meta-analysis detected 0.40% of HS prevalence in the analyzed countries. However, the authors observed that in studies based on direct evidence of clinical findings, the frequency of HS raised to 1.7%; being, therefore, higher than the values revealed by population-based analyses (Jfri et al., 2021).

Several clinical scores have been used so far to classify the severity of the disease: the Hurley classification, the modified Sartorius score (MSS), the HS Physician’s Global Assessment (HS-PGA), and the most recent score proposed the International Hidradenitis Suppurativa Severity Score System (IHS4) (Zouboulis et al., 2017). However, to date the simplest classification used is the Hurley, which stratifies the severity in three stages (Hurley I: mild; Hurley II: intermediate; Hurley III: severe) (Hurley, 1989). Because of the disease’s chronicity, debilitation, and heterogeneity, HS requires different treatment approaches that vary widely depending on disease severity (Costa-Silva et al., 2018). Unfortunately, to date, HS is considered a refractory disease. Drug therapy for HS patients comprises topical and systemic approaches, including prescription of anti-inflammatories and antibiotics depending on the Hurley stage and severity (Table 1).

**TABLE 1:** List of therapeutic categories and examples of available drugs with their main effect and indication to specific stages considering the Hurley stage classification.

Therapy category	Drug name	Main effects	Hurley stage
**Topical**	Clindamycin	Antibiotic/Anti-inflammatory	I
	Resorcinol	Keratolytic	I and II
	Fusidic acid	Antibiotic/Anti-inflammatory	I
**Systemic**	Clindamycin	Antibiotic/Anti-inflammatory	II and III
	Rifampicin	Antibiotic/	II and III
		Anti-inflammatory	
	Colchicine	Antibiotic/Anti-inflammatory	II and III
	Doxycycline	Antibiotic/Anti-inflammatory	I, II, and III
	Lymecycline	Antibiotic	I, II, and III
	Adalimumab	Anti-inflammatory (anti-TNF)	II and III
	Tetracycline	Antibiotic/Anti-inflammatory	I, II, and III
	Infliximab	Anti-inflammatory (anti-TNF)	II and III
	Minocycline	Antibiotic/Anti-inflammatory	II and III

Topical treatment is directed to mild and moderate cases (i.e., Hurley stages I and II). Clindamycin lotion is the most common treatment for early stage cases (Magalhães et al., 2019; Zouboulis et al., 2019; Sabat et al., 2020). However, other treatments have been considered for topical use based on positive clinical findings observed in HS patients, such as fusidic acid, resorcinol, an exfoliating agent with keratolytic, antipruritic, and antiseptic properties (Kilic et al., 2002; Wu et al., 2018; Magalhães et al., 2019).

In systemic therapy, antibiotics remain in clinical protocols with Clindamycin among the commonly used drugs (Ochi et al., 2018). Other first-line drugs used in systemic therapy employ tetracyclines and similar antibiotics, which were preferred by specialists to treat moderate to severe HS cases, except in children younger than 10 years old (Magalhães et al., 2019). Also, physicians observed a decrease in Sartorius score and other distress/pain indicators in HS patients who did not discontinue the treatment with tetracycline (*n* = 32), doxycycline (*n* = 31), and lymecycline (*n* = 45) (Jørgensen et al., 2021). The prospective results of three treatments indicate no significant difference in improvement and side effects, including gastrointestinal, neurological and dermatological symptoms. The anti-inflammatory activity of the second-generation tetracyclines (such as doxycycline) is probably related to their inhibition of pro-inflammatory cytokines (IL-1β, IL-6, TNF-α, IFN γ) and chemokines (MCP-1, MIP-1α, MIP-1β) synthesis (Krakauer and Buckley, 2003).

Once antibiotics were not successful to treat HS, biological drugs (based on antibodies targeting inflammatory cytokines) came as a potential alternative solution, including, for instance, Adalimumab and Infliximab. Adalimumab is the first fully human recombinant IgG monoclonal antibody that binds soluble and transmembrane TNF-α (Schneider-Burrus et al., 2018; Magalhães et al., 2019). It was approved by the European Medicine Agency and US Food and Drug Administration (FDA) as the first medicine for the treatment of moderate to severe HS patients, which failed in antibiotic (systemic) therapy (Schneider-Burrus et al., 2018).

It is important to emphasize that the improvement observed when using the biological drug treatment can be enhanced by combining it with antibiotics in topical or systemic therapy. Antibacterial drugs could play important roles in the topic treatment of HS also due to their complementary anti-inflammatory activities. Nonetheless, the predominance of antibiotic treatments can raise issues of toxicity and alarming resistant bacteria. Unfortunately, to date, data regarding bacteria resistance rates to antibiotic treatments in HS are scarce (Nikolakis et al., 2015; Fischer et al., 2017).

Considering this risk, the combination of antibiotics, including Clindamycin-Rifampicin treatment, was suggested as a preventive solution to overcome bacterial resistance over therapy (Ochi et al., 2018; Tyers and Wright, 2019). In addition to this approach, studies involving the prospection of novel drugs have been sought, for example, natural AMPs from distinct sources as scaffolds for drug design (Santos-Silva et al., 2020).

### Quality of Life and Prognosis

When considering the patients’ quality of life, painful lesions represent the main problem, being frequent and periodic, not always responding to a topic antibiotic therapy, aggravated with the onset of antibiotic bacterial resistance. So, the main questions are: how to manage these chronic inflamed wounds, how to treat them, how to fight pathogens infecting the pre-existing lesions? To face these issues, we should bear in mind that innate immune defects are known to play a role in HS etiopathogenesis, including host AMPs encoding genes that may be differentially expressed in wounds of patients suffering from HS. This issue will be further discussed.

## Antimicrobial Peptides

In the attempt to design novel strategies to fight pathogens infecting HS patient’s lesions and relieve the painful effect of the wounds, we suggest introducing novel players in this struggle to solve, at least partly, the patients’ lesion-related problems by employing antimicrobial molecules derived from plants. The use of natural compounds to limit infections by pathogens has been attempted in the context of different diseases such as psoriasis (natural compounds such as mustard seed, curcumin and resveratrol on inhibit NF-κB activation and cytokine expression, as well *Antrodia cinnamomea* extract to inhibit inflammatory cytokine expression) (Lai et al., 2017); rosacea (*Aloe vera* aloin, aloe emodin, aletinic acid, choline, and salicylate derivative) have been used to inhibit cyclooxygenase pathway; polyphenols contained in green tea such as epicatechin, epicatechin-3-gallate, epigallocatechin and epigallocatechin-3-gallate reduce ultraviolet B induced inflammation by functioning as an antioxidant (Draelos, 2017); chronic diseases have been targeted by flavonoids with effects on inflammasome regulation and catechin with antioxidant action (Owona et al., 2020); skin infections have been treated with Nigritanine, isolated from *Strychnos nigritana*, characterized by antimicrobial activity against clinical isolates of *S. aureus* showing a remarkable antimicrobial activity, without being toxic *in vitro* to mammalian red blood cells and human keratinocytes; this latter compound can be considered as a promising candidate for the development of new antimicrobial molecules for the treatment of *S. aureus* induced infections (Casciaro et al., 2019). Nevertheless, most of the studies just used crude extracts from plants (leaves, roots, bark, etc.) without succeeding to clearly identify the antimicrobial molecule/s involved in limiting pathogens infection. Moreover, the laboratory pipeline to identify and characterize antimicrobial molecules is time-consuming since chromatography (HPLC) and Mass Spectrometry techniques should be used. Finally, secondary metabolites in the crude extracts can mask the effective molecule responsible for the antimicrobic activity. These limitations can be circumvented using computational tools in the search for the most suitable bioactive molecules, since it is possible to identify and isolate these molecules through data mining, as well as predict their respective activities and cytotoxicity even before solid-phase synthesis or heterologous expression. These steps are shown in more detail in topic 4 Bioinformatics toward new synthetic compounds.

### General Information

Antimicrobial peptides, also called host defense peptides, are evolutionarily conserved structures present in most living organisms, participating in the innate immune system (Si-Tahar et al., 2009).

In eukaryotes, these peptides act predominantly in intercellular communication, with roles that interfere with hormones, growth factors, and the defense system (Marmiroli and Maestri, 2014). In this sense, in mammals, AMPs are recognized as the first protective barrier against pathogens (de la Fuente-Núñez et al., 2017) present, therefore, in the skin, mucous membranes, and systems such as the urogenital, respiratory, or gastrointestinal (Xia et al., 2018). These molecules often show significant diversity in their size and amino acid composition; however, they share three-dimensional structural characteristics (Kosikowska and Lesner, 2016). Although sometimes linear, they can manifest polycyclic structural diversity such as lantibiotics in Gram-positive bacteria, cyclotides in plants, and theta-defensins in animals (Montalbán-López et al., 2012). These features are responsible for the dynamic nature of these molecules and the electrostatic attraction between cationic peptides and the anionic surface of bacterial membranes rich in phosphatidylglycerol, culminating in membrane lysis. Interestingly, these same cationic peptides do not usually affect mammalian cell membranes, rich in zwitterionic phospholipids, whose charge is neutral (Kosikowska and Lesner, 2016; Kang et al., 2017). These attributes give the AMPs a broad spectrum of activities against bacteria, yeasts, viruses, and fungi, in addition to performing wound healing and cytotoxic effects on cancer cells, besides exercising immunomodulatory activities (Baxter et al., 2017; Kang et al., 2017). However, most AMPs identified so far have only been tested *in vitro* against fungi and bacteria (Bastos et al., 2018), with a great demand for *in vivo* studies and clinical tests.

Even scarce, *in vivo* trials with mice generated promising results, demonstrating the antimicrobial effects of these AMPs, limiting microbial proliferation on surfaces and mucous membranes, thus preventing dissemination to deep tissues and consequent worsening of the infection (Nizet, 2006).

### Antimicrobial Peptides in Health Human Skin

To understand the strong antimicrobial activity of AMPs, we can consider that although human skin, which is protected primarily by the keratinized cells of the outer layers, is constantly exposed to microorganisms, it rarely becomes infected; AMPs are one of the primary mechanisms used by the skin in the early stages of immune defense (Bardan et al., 2004).

Antimicrobial peptides present in the human skin are principally defensins (human β-defensins 1–3), cathelicidins (LL-37), dermcidin (DCD), and other small peptides such as proteinase inhibitors, chemokines and neuronal proteins/peptides with RNase 7 and psoriasin (S100A7) (Bardan et al., 2004; Yamasaki and Gallo, 2008). Several functionalities are attributed to them, ranging from cell migration and differentiation, to immunomodulatory activity, acting in the control and production of mediators such as cytokines and chemokines, helping in wound healing (Ring et al., 2018). However, several studies indicate that defects in the regulation of these molecules can actively contribute to the pathogenesis of various skin diseases such as acne vulgaris, atopic dermatitis (AD), psoriasis and HS (Mangoni et al., 2016; Niyonsaba et al., 2017; Herman and Herman, 2019).

These AMPs are produced by different cell types present in the skin, particularly keratinocytes, sebocytes, neutrophils, and eccrine sweat glands (Schittek et al., 2008). Most AMPs are constitutively secreted at the surface of the skin but also increase after various stimuli, including microorganisms, cytokines, and injury (Mangoni et al., 2016). In particular, AMPs expression is associated with tissue differentiation (Heilborn et al., 2003), wound healing, and skin recovery in a bidirectional way, simultaneously acting to combat the pathogens proliferation and biofilm formation in damaged tissue (Mangoni et al., 2016). Besides, AMPs have a role in modulating cytokine production, cell proliferation, and angiogenesis (Steinstraesser et al., 2008; Otvos and Ostorhazi, 2015).

### Antimicrobial Peptides in Hidradenitis Suppurativa

Altered expression of AMPs has been observed in some skin diseases, including HS. Schlapbach et al. (2009), detected an overexpression of mRNA and protein levels of psoriasin and hBD-2 mRNA in HS lesions compared with normal skin; instead, the hBD-2 protein level was significantly lower in HS skin compared to normal skin (Schlapbach et al., 2009). Wolk et al. (2011) found a deficiency of several AMPs (BD-1, BD-2, BD-3, S100A7, S100A8, and S100A9) in HS lesions (Wolk et al., 2011). Hofmann et al. (2012) observed a decrease in the expression of these peptides, in particular hBD-3, in HS patients versus normal controls, which may predispose them to the observed greater susceptibility to infections with formation of skin lesions, aggravating the skin condition (Hofmann et al., 2012). In fact, the activity of AMPs associated with their tissue expression is closely linked to the degree of pathogenic infections in skin lesions. Thus, it is understood that this interaction throughout evolution resulted in the relevance of AMPs as regulators and modulators of the skin microbiota and as the main responsible for the defense and maintenance of skin health (Schauber and Gallo, 2008; Herman and Herman, 2019).

### Plant Antimicrobial Peptides

In plants, AMPs are part of the immune system and exhibit broad-spectrum antimicrobial activity as their main characteristic (Li et al., 2021). After sensing a pathogen, a signaling cascade is triggered, inducing the strengthening of the cell wall, the production of secondary metabolites such as phytoalexins, tannins, polyphenolic compounds, and the synthesis of pathogenesis-related (PR) proteins (Benko-Iseppon et al., 2010). These PR proteins were first discovered in the early 1970s in tobacco in response to mosaic virus infection and later classified according to their response to pathogen attacks (Ebrahim et al., 2011; Sinha et al., 2014).

Although plant AMPs have been extensively studied, a recent publication by Petre (2020) raised a discussion regarding the concept of host-defense peptides (HDPs), which are bifunctional peptides with direct antimicrobial and immunomodulatory activities. In this perspective article, a list of six defense peptides (HDP candidates) was identified, thanks to literature survey (MsDef1, DEF2, IbACP, PdPR5-1, RISP, and CaAMP1). These candidates, in addition to their already known antimicrobial activity, also have the ability to alter the physiology of the plant in a way that suggests a potential role as immunomodulators. To date, there is little information oh HDPs, but fortunately these results indicates that HDPs can be detected in already well characterized and conserved plant AMP gene families, facilitating future functional investigations.

Plant AMPs classification is based mainly on their primary (linear) and three-dimensional structures, with eight AMP families prevailing (Table 2): Thionins, Defensins, Hevein-like, Knottin-Type, α-Hairpinins, Lipid Transfer Proteins, Snakins, and Cyclotides (Li et al., 2021).

**TABLE 2:** Main classes and features of plant antimicrobial peptides.

General information							Primary structure		Tridimensional structure	
Class	Biological roles and action mechanism ^(A)^	Number of cysteines and disulfide (S-S) bridges ^(B)^	UniProt – ID^(C)^	AMP name^(D)^	Source species^(E)^	Mass (Da) ^(F)^	Length^(G)^	Domain^(H)^	General structural motif^(I)^	General 3D – structure^(J)^
Thionins	Antimicrobial activity and signal transduction. Molecules probably change plasma membranes potential inducing efflux of K+ and Ca2+ influx.	6C = C1–C6, C2–C5, C3–C4	P01542	Crambin	*Crambe hispanica*	4,736	46	1–46	β1-α1-α2-β2-coil	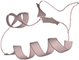
		8C = C1–C8, C2–C7, C3–C6, C4–C5	P01543	Purothionin A-1	*Triticum aestivum*	14,625	136	28-72		
Defensins	Antimicrobial activity. Molecules probably change plasma membranes potential inducing efflux of K+ and Ca2+ influx.	8C = C1–C8, C2–C5, C3–C6, C4–C7	P30230	Defensin-like protein 2	*Raphanus raphanistrum*	8,875	80	30–80	CSαβ (β1-coil-α-β2-β3) motif	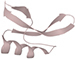
		10C = C1–C10, C2–C5, C3–C7, C4–C8, C6–C9	Q8H6Q1	Defensin-like protein 1	*Petunia hybrida*	11,361	103	26–72		
Hevein-Like	Antimicrobial activity. Molecules probably target: *N*-acetyl-glicosamine, but also, has inhibitory activity described against non-chitin fungus.	6C = C1–C4, C2–C5, C3–C6,	Q9S8Z6	AC-AMP1	*Amaranthus caudatus*	3,034	29	1–29	Gly and Cys rich Central β-strands and short helical side coils	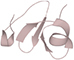
		8C = C1–C4, C2–C5, C3–C6, C7–C8	P02877	Pro-hevein	*Hevea brasiliensis*	21,859	204	18–60		
		10C = C1–C5, C2–C9, C3–C6, C4–C7, C8–C10	P85966	Antimicrobial peptide 1b	*Triticum kiharae*	11,46	116	35–78		
Knottin-type peptide	Antimicrobial activity and protease inhibitor. Probably target: plasma membrane, α-amylase or protease inhibitors (carboxypeptidase A or trypsin)	6C = C1–C4, C2–C5, C3–C6,	P83653	Antimicrobial peptide Alo-3	*Acrocinus longimanus*	3,875	36	1–36	Cystine knot (Short β-strand and coil)	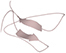
α-Hairpinin	Antimicrobial activity. Probably target: Intracellular DNA or RNA. It also has trypsin inhibitory, ribossomal inactivation activities described.	4C = C1–C4, C2–C3	R4ZAN8	L-2	*Triticum kiharae*	41,467	362	125–152	α1-turn-α2	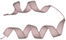
Lipid transfer proteins	Antimicrobial activity and transport hydrophobic molecules, such as fatty acids. Probably target: plasma membrane, acetyl-CoA and posphatidyl glycerol	8C = C1–C6, C2–C3, C4–C7, C5–C8	Q0IQK9	Non-specific lipid-transfer protein.	*Oryza sativa* subsp. *japonica*	11,345	116	26–116	Hydrophobic cavity (α1-α2-α3-α4-coil)	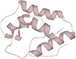
Snakin	Antimicrobial activity Probably target: plasma membrane and chitin.	6C = C1–C9, C2–C7, C3–C4, C5–C11, C6–C12, C8–C10	Q948Z4	Snakin-1	*Solanum tuberosum*	9,664	88	29–88	α -helix	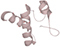
Cyclotide	Antimicrobial and protease inhibitor activities Probably target: plasma membrane and trypsin.	3C = C1–C4, C2–C5, C3–C6	P84522	Leaf cyclotide 1	*Viola hederacea*	3,341	31	4–31	Cyclic cystine knot (CCK)	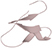
β-barrelins	Antimicrobial activity. Probably target: plasma membrane.	6C = C1–C5, C2–C6, C3–C4	P80915	Antimicrobial peptide 1	*Macadamia integrifolia*	10,944	102	27–102	Greek key β-barrel	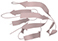
Impatiens-like	Antimicrobial activity. Probably target: Targets either membrane and/or intracellular components.	4C = C1–C3, C2–C4	O24006	Antimicrobial peptides	*Impatiens balsamina*	37,259	333		β-turn	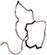
Puroindoline	Antimicrobial activity. Targets membrane.	10C = C1–C7, C2–C5, C3–C4, C6–C10, C8–C9	P33432	Puroindoline-A	*Triticum aestivum*	16,387	148	29–146	Tryptophan pocket (α1-α2-α3-α4)	Structures not found
Thaumatin-like	Antimicrobial Activity. Targets plasma membrane.	16C = C1–C16, C2–C3, C4–C5, C6–C15, C7–C14, C8–C9, C10–C11, C12–C13	G5DC91	Thaumatin-like protein 1	*Manilkara zapota*	21,922	207	1–207	Acid cleft (REDDD)	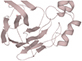

*In (A) probably roles and mechanism of action. In (B) the number of cysteines and disulfide bonds formation. In (C) identifier in the UniProt database. In (D) common name. In (E) source species (first description). In (F) mass in Kilo-Daltons. In (G) length of peptide. In (H) domain of the mature peptide. In (I) generic structural motif. In (J) three-dimensional representation.*

As highlighted by Santos-Silva et al. (2020), plant AMPs have greater diversity and abundance compared to AMPs from members of other kingdoms. This is probably related to the sessile nature of plants associated with environmental evolutionary forces, in addition to their redundant genomes. Therefore, it can be speculated that plants harbor many AMPs not yet described, given their vast abundance and diversity of forms. In fact, a single plant can contain a wide variety of AMPs as an arsenal for the diversity of pathogens found (Noonan et al., 2017).

In recent decades, several studies have been published regarding the potential of these plant AMPs. Porto et al. (2018a) compared the anti-infective potential of guavanin 2 as compared with its ancestors (Pg-AMP1 and fragment2), using the ‘abscess skin infection model’ with *P. aeruginosa*. All three AMPs in question presented activity against this bacterium, which can cause skin infections in healthy or immunocompromised patients (Pelegrini et al., 2008).

Other studies reported the prospective use of AMPs against various bacteria (Fensterseifer et al., 2015) and fungi (Maracahipes et al., 2019; dos Santos-Silva et al., 2021), indicating their potential as a new therapeutic approach in the treatment of local and systemic infections, including those against bacteria multi-resistant to antibiotics (Almaaytah et al., 2017). The authors highlighted the need to use robust methodologies to identify and circumvent the possible side effects that may appear in the interactions of these molecules.

## Bioinformatics Toward New Synthetic Compounds

The scarcity of effective and affordable drugs available worldwide is a severe problem in modern society, preventing the treatment of some diseases and eradicating infectious agents. Furthermore, the rapid spread of pathogens resistant to the existing drugs increases the global public health crisis, being a challenge for modern medicine to find alternative therapeutic options that can deal with increasingly resistant pathogens (O’Neal et al., 2020). In this scenario, plants emerge as an almost inexhaustible and still little explored reservoir of new drug candidates to fight various types of diseases (Broni et al., 2021). Given such abundant resources, the interest to develop new methodologies to analyze these biomolecules and explore the molecular treasures of plant species has increased.

Although traditional organic chemistry is a powerful tool to find new therapeutic and medicinal biomolecules, the search for compounds with the aid of bioinformatics emerges as an alternative and possibly advantageous option. It helps to reduce efforts and time, significantly decreasing the number of target compounds and filtering out only the best candidates for *in vitro* tests (Barrera-Vázquez et al., 2021). Besides, the current great availability of sequenced omics (e.g., genomes transcriptomes proteomes, etc.) data has undoubtedly opened new paths for innovative projects using informatic technology tools in search of new therapeutic molecules for synthetic biology (Broni et al., 2021), also applicable to AMPs (Santos-Silva et al., 2020; dos Santos-Silva et al., 2021).

**FIGURE 1:**
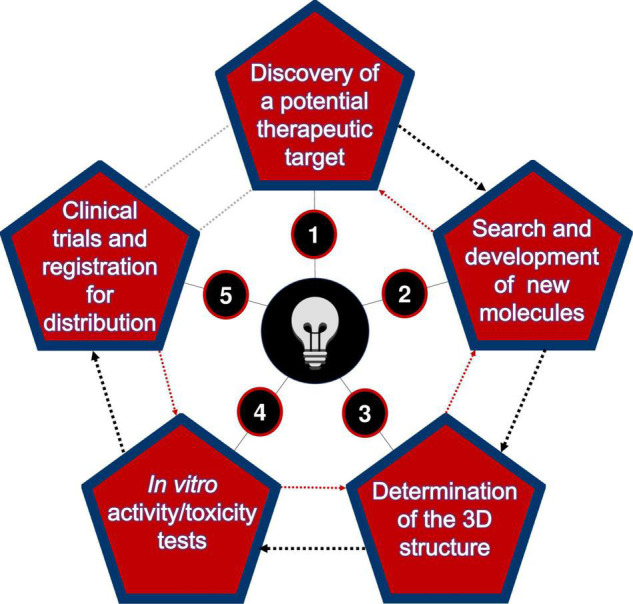
Main milestones to be overcome in the search for new therapeutic compounds.

Generally, the discovery process of new biomolecules consists of the following main steps (Figure 1). The first is the discovery phase, where a potential therapeutic target is identified. At this stage, a systematic literature search is needed to know all the possible interactions that the targets may perform with other (already discovered) molecules (Kantardjieff and Rupp, 2004).

In the second step, once the specific target has been identified, several potential interacting molecules can be searched among those existing in databases. It is also possible to create molecules with discrete modifications based on computational predictors (Porto et al., 2018a). It is also possible to simulate the binding of these molecules with the chosen target, thus performing the virtual screening assay (Graff et al., 2021), thus helping to model the failure potential for the subsequent phases (Katara, 2013). It is important to note that most prediction tools are based on machine learning and predictive modeling techniques to increase the potential to generate useful information to develop a new drug (Porto et al., 2018b).

The third phase consists of determining the three-dimensional structure of the candidates selected in the previous step, either by wet-lab approaches [as X-ray crystallography, nuclear magnetic resonance (NMR), or electronic microscopy] or by computational (*in silico*) predictive methods such as comparative three-dimensional modeling approaches, *threading* or *ab initio* (Lee J. et al., 2017). Given their feasibility and speed, *in silico* methods have been the most used, benefiting from the increasing accuracy of prediction algorithms, access to different aspects of AMP structural complexity, and low costs to solve certain structures (Santos-Silva et al., 2020). In this scenario, recent results in structural biology have indicated that the folding of distinct proteins is limited (Wang et al., 2021). Contrary to original expectations that many new structures (and folding domains) would soon be discovered, it has been recognized that biological relevance, design, and diversity of these biomolecules are relatively conserved (Breinbauer et al., 2003). This means that the same structural domain can be used by different proteins with some modifications (Santos-Silva et al., 2020).

The fourth phase demands *in vitro* and *in vivo* (animal models) testing to determine the efficacy of the compounds’ activity and their cytotoxicity and organ toxicity potential, aiming to proceed to the fifth phase, the clinical trials and production licensing aimed at further distribution in the market for clinical use. The high cost of this phase acts as a limiting factor for the number of drugs that pharmaceutical companies or public institutions can develop; therefore, selecting the compounds with the best chances of approval is critical (Batool et al., 2019). Although complex and time-consuming, this step is potentially rewarding. The development of a new drug requires technological expertise, human and financial resources. It also requires strict compliance with regulations on testing standards, ethical aspects, and manufacturing before this new drug enters the market and can be used in the general population (Katara, 2013).

It is noteworthy that the steps reported in Figure 1 do not obligatorily follow a single direction. Several times it becomes necessary to go back to a previous phase, since for some reason, the most promising molecule may not proceed to the subsequent phases, due to some unwanted effect, such as high toxicity during *in vitro* or *in vivo* trials. All subsequent efforts, whether for small molecule identification, compound optimization, pharmacokinetic studies, or a clinical trial, will be as effective as the initial decision to choose one target or another (Malempati et al., 2012; Basith et al., 2020). It should also be noted that predictors cannot simulate such complex biological systems, even with improvements being made almost daily. Thus, there will always be a demand for new, improved tools or alternative ways to predict novel effective molecules (Patel et al., 2021).

Another promising approach involves the development ‘by design’ of selective and effective peptides to treat specific diseases (Kantardjieff and Rupp, 2004). The knowledge of disease-related genetic biomarkers guides pharmaceutical companies to design more accurate and individualized drugs and dosages based on pharmacogenomics (Liou et al., 2012; Hirtz et al., 2021). Such an approach allows drugs to be developed that meet the requirements of specific genetic subgroups of the general population (Nelson et al., 2009), allowing the prescription of drugs based on the genetic profiles of their patients, the so-called “Personalized Medicine.” This is an emerging area, which has been growing more and more in recent decades, to improve patients’ treatment and prognosis. However, several uncertainties remain about how to fulfill the task of maximizing benefits and reducing risks and how to select patients who can benefit from a particular drug (Chang et al., 2021).

## Peptide Therapeutics: Preclinical and Clinical Trials

Recent (August 2021) reports on the AMP repository (DRAMP^1^) show that about 77 peptides are in the last stages of development to be used as new pharmaceutical agents (Shi et al., 2021). Despite their potential, only a limited number of AMPs are available as regularly registered drugs. However, this number is expected to grow, as around 155 drugs of a peptide nature have been tested, and just under half are currently in Phase 2 clinical trials (Lau and Dunn, 2018; Schultz et al., 2019; Vilela et al., 2021). In 2020, the Food and Drug Administration (FDA) approved 53 new drugs, six of which are peptides and oligonucleotides. Besides, along 2016–2020 a total of 14 peptides have been approved as new drugs. These numbers further strengthen the market for peptides which, numerically, represent about 10% of the total approved drugs. Despite the promising numbers, it is noteworthy that scientific research in recent years was impacted by the COVID-19 pandemic, which interfered in several aspects of the development and approval of new drugs, even those not related to this infectious disease (Al Musaimi et al., 2021).

The growing development of new peptide-based drugs has driven intense research and investment in these molecules by diverse academic and industrial groups over more than 30 years, consequently increasing the rate of clinical trials (de la Torre and Albericio, 2020). However, unfavorable physicochemical properties, such as high molecular weight, susceptibility to digestive enzymes, hydrophilicity, and low intestinal permeability, attenuate the effects of these peptides, preventing their approval after clinical trials (Aguirre et al., 2016).

Furthermore, *in vitro* microbial inhibitory concentration (MIC) values sometimes do not correlate with *in vivo* efficacy due to the loss of electrostatic interactions between the peptides and the cell membrane. Besides, most AMPs bind to serum proteins, such as albumin and lipoproteins, which decreases their effectiveness against pathogens. Therefore, AMPs should be tested in different media to validate their effectiveness (Koo and Seo, 2019).

Moreover, it is known that peptides in general can have cytotoxic effects if administered in oral supplementation or intravenously. Oral administration has been inadvisable due to a possible loss of membrane selection and toxicity. For other drugs, such problems have been solved through technological advances like encapsulation or slow release.

Despite these limitations, AMPs have several advantages, such as broad-spectrum antimicrobial activity, even against multi-resistant pathogens. These molecules are highly effective against gram-negative bacteria, considered more challenging targets than gram-positive bacteria that present a relatively lower probability of developing resistance (Koo and Seo, 2019).

These peptides can vary in size (generally 5–44 amino acid residues), structure, and indication for use, including, for example, from specific targets in combating diabetes, HIV, cystic fibrosis, obesity reduction, and in most cases to fight infections (Al Musaimi et al., 2021). Table 3 compiles examples of Peptide-based Drugs that have passed clinical trials. The approval of these peptides represents an incentive for innovation in the production of medicines and the inclusion of alternative therapies in the treatment of rare diseases more safely and efficiently (de la Torre and Albericio, 2020).

**TABLE 3:** Peptide-based drugs approved by the Food Drug Administration (FDA) (2015–2020) (de la Torre and Albericio, 2020; Al Musaimi et al., 2021).

Trade name	Indication	Year
Insulin degludec Tresiba^®^	Diabetes	2015
Ixazomib Ninlar^®^	Multiple myeloma	2015
Adlyxin^®^ Lixisenatide	Diabetes	2016
Abaloparatide Tymlos^®^	Osteoporosis	2017
Angiotensin II Giapreza^®^	Hypotension	2017
Etelcalcetide Parsabiv^®^	Hyperparathyroidism	2017
Macimorelin Macrilen^®^	Growth hormone deficiency	2017
Plecanatide Trulance^®^	Chronic idiopathic constipation	2017
Semaglutide Ozempic^®^	Diabetes	2017
^177^Lu DOTA-TATE Lutathera^®^	Neuroendocrine tumors, theragnostic	2018
^68^Ga DOTA-TOC Afamelanotide	Neuroendocrine tumors, diagnostic	2019
Scenesse^®^ Bremelanotide	Skin damage and pain	2019
Vyleesi^®^ Enfortumab Vedotin-Ejfv	Women hypoactive sexual desire	2019
PADCEV^®^ Polatuzumab Vedotin-Piiq	Cancers expressing Nectin-4	2019
Polivy^®^	Diffuse large B-cell lymphoma	2019
Setmelanotide (ImcivreeTM)	Obesity	2020
[_64_Cu]-DOTATATE (DetectnetTM)	PET imaging	2020
[^68^Ga]-PSMA-11	Diagnosis of recurrent prostate carcinoma by PET	2020
Mafodotin-blmf (Blenrep™)	Relapsed or refractory multiple myeloma	2020

Koo and Seo (2019) listed 36 promising AMPs, 27 in clinical trials, and 10 in preclinical stages. One of them (D2A21) is included in two different trials for different indications (skin infections and burn wound infections) (Table 4). The authors report that information about other peptides could not be obtained because licenses were transferred to other companies, or preclinical or clinical trials were stopped for unknown reasons. To decrease the chances of failure, the targeting and therapeutic peptide selection process must go through an extensive protocol long before it is synthesized, as discussed above.

**TABLE 4:** Antimicrobial peptides in preclinical and clinical trials (phase I–III) (Koo and Seo, 2019).

AMP	Target	Phase
EA-230	Sepsis and renal failure protection	II
CZEN-002	Anti-fungal	II
D2A21	Burn wound infections	III
XMP-629	Impetigo and acne rosacea	III
Neuprex(rBPI21)	Pediatric meningococcemia	III
Delmitide(RDP58)	Inflammatory bowel disease	II
Ghrelin	Chronic respiratory failure	II
NVB-302	*Clostridioides difficile*	I
hLF1-11	MRSA, *K. pneumoniae, L. monocytogenes*	I/II
Wap-8294A2 (Lotilibcin)	G(+) bacteria(VRE, MRSA)	I/II
C16G2	Tooth decay by *Streptococcus mutans*	II
SGX942(Dusquetide)	Oral mucositis	III
DPK-060	Acute external otitis	II
PXL01	Postsurgical adhesions	III
PAC113	Oral candidiasis	II
POL7080	*Pseudomonas aeruginosa*, *Klebsiella pneumoniae*	III
LTX-109 (Lytixar)	G(+) MRSA skin infections, impetigo	II
OP-145	Chronic middle ear infection	II
LL-37	Leg ulcer	II
Novexatin (NP213)	Fungal nail infection	II
p2TA (AB103)	Necrotizing soft tissue infections	III
Iseganan (IB-367)	Pneumonia, stomatitis	III
Pexiganan (MSI-78)	Diabetic foot ulcers	III
Omiganan (CLS001)	Rosacea	III
Surotomycin	*Clostridioides difficile* (diarrhea)	III
Ramoplanin (NTI-851)	G(+) (VRE, *Clostridioides difficile*)	III
Friulimicin B	Pneumonia, MRSA	I
MU1140	G(+) bacteria (MRSA, *Clostridioides difficile*)	Preclinical
D2A21	Skin infections *(Pseudomonas aeruginosa, Staphylococcus aureus*)	Preclinical
HB1275	Fungal skin infections	Preclinical
HB1345	Skin infections, acne	Preclinical
Arenicin (AP139)	G(-) bacteria, UTI	Preclinical
AP114	*Clostridioides difficile*	Preclinical
AP138	MRSA	Preclinical
Novamycin (NP339)	Fungal infections	Preclinical
Novarifyn (NP432)	Broad-spectrum G(+) and G(-)	Preclinical
Avidocin and Purocin	G(-) bacteria	Preclinical

It is noteworthy that several AMPs in advanced clinical and preclinical stages have applications in infectious and inflammatory skin diseases (Table 4), such as impetigo, acne, rosacea, different skin infections, and burn wound infections, among others. However, peptide design techniques require skills and knowledge in several areas, especially in biophysics, chemistry, statistics, and informatics. There is no single way to carry out peptide design, although some approaches have been used with greater success, as follows.

– Structure-activity relationship (SAR): in this strategy, the objective is to maximize antimicrobial activity and resistance to proteolytic degradation while minimizing toxicity to the host, considering modeling as an important component for better integrating physicochemical and biological properties (Liu et al., 2020).
– Site-directed mutagenesis: this approach involves reengineering natural peptides by adding, deleting, or replacing one or a few amino acid residues. It aims at identifying peptide variants with improved activity, finding specific positions in the sequence where the mutation is beneficial for the antimicrobial activity (Bozovičar and Bratkovič, 2019). Some techniques can support this approach, such as alanine or lysine scanning, which are tools used to find mutable amino acids in the sequence with a low risk of damage to the three-dimensional structure (Gunasekera et al., 2018). Another technique is named “peptide shuffling,” in which the amino acid residues are shuffled to generate AMPs with the same composition but with residues in different orders. However, this method involves a more complex downstream analysis to isolate the sequence requirements associated with the desired function (Torres et al., 2019).
– *De novo* design: through this approach, peptides present basic and hydrophobic amino acid residues in a given sequence so that they exhibit hydrophobic and hydrophilic regions, favoring an amphipathic structure with general antimicrobial activity (Chen et al., 2019). Although this methodology is quite robust, it can generate peptides without membrane specificity. Besides, a newly generated peptide may even present a different three-dimensional structure and higher cytotoxicity (Zheng et al., 2019).
– Synthetic combinatory library: it is a powerful and widely used tool to obtain optimized classes of active peptides quickly. This approach generates tens to thousands of peptides with a defined structure, which can be confirmed by analytical lab techniques. Notably, this type of combinatorial approach should have a particular screening strategy depending on the target. Besides, the use of this approach has the disadvantage of the high cost of generating these libraries, which makes positional scanning or iterative approaches very expensive (Rognan, 2017; Naseri and Koffas, 2020). Although many researchers are looking for approaches that use smaller libraries of compounds with similar properties, for targets with little information available, high diversity library methods undoubtedly appear to be essential (Liu et al., 2017).
– Template-assisted approach: it is possible to extract patterns, motifs, or even domains from natural sequences, which can be used as inspiration to design a new peptide. It has the advantage of significantly reducing the costs of the most promising set of peptides that need to be synthesized. Furthermore, this type of approach has the advantage of keeping some evolutionary information focusing on conserved patterns, which could be lost by other approaches listed above (Torres et al., 2019; Poolakkandy and Menamparambath, 2020).
– Methods based on computational prediction: Known peptides available in databases can be used as models to predict AMP activity with the aid of predictive algorithms (as neural networks, genetic algorithms, machine, and deep learning) (Porto et al., 2018b). Such an approach requires in-depth knowledge of statistical mathematics associated with predictive computing strategies to recognize regions with potential antimicrobial activity. Such an approach can even be used in association with other methods to improve AMP activity (Waghu et al., 2016).

All methods described are instrumental, and the choice depends on the type of therapeutic target, besides the available computational and lab resources. All of them have limitations and depend on the skill of the researchers involved. Also, the combination of different techniques has proven to be the ideal way to design peptides, providing helpful information about their possible roles, making these approaches an almost inexhaustible source of possible candidate antibiotic peptides.

**FIGURE 2:**
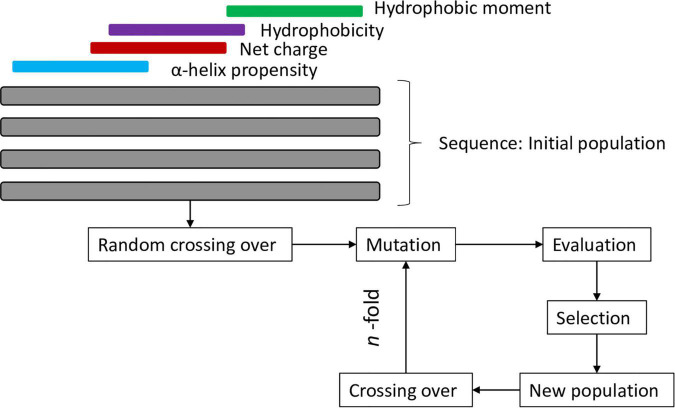
Flowchart of genetic algorithm of Porto et al. (2018a).

An example of the effective use of these methodologies is the guava peptide reported by Porto et al. (2018a) which was designed using a genetic algorithm that simulated the evolution of a population of sequences over a series of iterations. To select the best candidates, some important attributes were considered, such as the propensity for α-helix formation, the net positive charge, hydrophobic regions and the hydrophobic moment (the flowchart of the strategy of this design can be found in Figure 2). This peptide showed broad-spectrum antimicrobial activity including *S. aureus*, which is the most frequently found pathogen in human skin and wound infections.

In some cases, the design of peptides is usually simpler with the repetition of antimicrobial motifs, as the case with the study by Mohamed et al. (2016), which showed the antimicrobial activity of two peptides, WR12 (RWWRWWRRWWRR) and D-IK8 (IRIKIRIK), against multi-resistant *S. aureus*. This type of approach can present peptides with potent antibacterial activity, but it runs the risk of presenting low membrane selectivity, becoming toxic to the host’s cells. On the other hand, short and simplified sequences can facilitate its rapid production, in addition to lowering its synthesis cost and accelerating its clinical applications.

## Concluding Remarks and Perspectives

For all the reasons presented here, many AMPs are currently undergoing clinical trials, and the number is expected to increase even more due to the enormous potential these molecules have.

In the clinical practice in general, and specifically in the dermatological clinical practice related to HS, the need for new antibiotics or drugs with novel mechanisms of action is emerging. Additional effects, such as anti-inflammatory and immunomodulatory, are desirable, whereas few therapeutic drugs have all these attributes. In this scenario, plant AMPs figure as promising candidates for a new class of antibiotics.

We hypothesize that introducing plant derived AMPs, designed and engineered by using bioinformatic techniques could contribute to discover novel compounds to be possibly employed to improve the treatment of painful lesions affecting HS patients’ quality of life.

Most studies have applied traditional approaches involving the isolation of AMPs by organic chemistry techniques to carry out exploratory tests. The integration of large amounts of omics data with bioinformatics tools has opened new possibilities for design of a multitude of novel peptides with therapeutic potential. In this scenario, plants stand out for the richness and diversity of molecules they present. These approaches associated with peptide engineering allow the development of new “custom-made” molecules inspired by native compounds, with less chance of toxicity and off-target effects.

Finally, the computationally developed AMPs have several advantages over traditional antibiotics, among them we can include a broad spectrum of activity, low potential for resistance development, ability to neutralize virulence factors released by pathogens, ability to modulate the host’s immune response, all of this with the possibility of using these peptides as topical antibacterial agents that could be the future in the treatment of HS.

## Author Contributions

CS-S performed the literature review and wrote the manuscript. PT wrote the specific chapters and critically revised the text and included relevant suggestions. LV, RR-F, VA, Ad’A, and MR wrote the specific chapters. AB-I critically revised the text and included relevant suggestions. SC conceived the review, wrote the introduction, concluding remarks, and besides critically revising the manuscript. All the authors have read the manuscript and agreed to its content.

## Conflict of Interest

The authors declare that the research was conducted in the absence of any commercial or financial relationships that could be construed as a potential conflict of interest.

## Publisher’s Note

All claims expressed in this article are solely those of the authors and do not necessarily represent those of their affiliated organizations, or those of the publisher, the editors and the reviewers. Any product that may be evaluated in this article, or claim that may be made by its manufacturer, is not guaranteed or endorsed by the publisher.

## Footnotes

1
http://dramp.cpu-bioinfor.org/
